# China's Growing Contribution to Global Intracranial Aneurysm Research (1991–2012): A Bibliometric Study

**DOI:** 10.1371/journal.pone.0091594

**Published:** 2014-03-12

**Authors:** Ze-jun Jia, Bo Hong, Da-ming Chen, Qing-hai Huang, Zhi-gang Yang, Cha Yin, Xiao-qun Deng, Jian-min Liu

**Affiliations:** 1 Department of Neurosurgery, Changhai Hospital, Second Military Medical University, Shanghai, China; 2 Editorial Department of Academic Journal of Second Military Medical University, Department of Research, Second Military Medical University, Shanghai, China; 3 Shanghai Information Center for Life Science, Shanghai Institutes for Biological Sciences(SIBS), Chinese Academy of Sciences(CAS), Shanghai, China; Max Planck Society, Germany

## Abstract

**Background:**

We sought to analyze the growing worldwide trends of intracranial aneurysm research, investigate China's recent contribution, and compare the contributions of mainland China, Taiwan, and Hong Kong.

**Methods:**

Global and China intracranial aneurysm-related publications were retrieved from the Web of Science database from 1991 to 2012. Excel 2007, Matlab, and Thomson Data Analyzer (TDA) software were used to analyze the search results for number of publications, cited frequency, h-index, and organization contributions.

**Results:**

16468 global papers were identified that were cited 273500 times until 2013-08-15. The United States accounted for 31.497% of the articles, 58.64% of the citations, and the highest h-index (127). Japan and Germany followed in frequency. China's articles ranked eighth (third in 2012) in total number, with most of the contributions occurring since 2002 (91.33%). China was at the early stage of the logic growth curve (exponential growth), with the citation frequency and h-index per year increasing. The quality of the publications was low. The main research centers were located in Beijing, Shanghai, Taiwan, and Hong Kong. The main Asian funding body was the National Natural Science Foundation of China. The number of publications and frequency of citations of papers from mainland China was greater than that of Taiwan or Hong Kong.

**Conclusion:**

Global intracranial aneurysm research has been developing swiftly since 1991, with the United States making the largest contribution. Research in China started later, in 2002. Since then, China has increased its rate of publication, and became the third largest contributor by 2012.

## Introduction

Hemorrhagic stroke caused by intracranial aneurysm rupture is one of the most threatening cerebrovascular diseases. It is characterized by high disability and fatality, and is a focus of neurosurgery today[1]. Guglielmi detachable coils, first contrived in 1991, marked a new era for endovascular interventional treatment of intracranial aneurysms[2], [3]. The International Subarachnoid Aneurysm Trial (ISAT) study published in 2002 confirmed that endovascular interventional treatment was superior to craniotomy occlusion in the treatment of intracranial aneurysms, further accelerating the study of intracranial aneurysms[4]. With the improvement of the overall economy and scientific research strength in China[5], [6], rapid progress has also been seen in Chinese clinical and basic research of intracranial aneurysms during the past two decades. The global and China's development momentum regarding intracranial aneurysms has not been well studied.

Bibliometrics, using the literature system and literature metrology characteristics as research objects and analyzing the literatures quantitatively and qualitatively, can help characterize development in a field, predict trends in development, provide an auxiliary basis for identifying research projects, and guide clinical and basic medical research[7], [8]. Remarkable progress has been achieved in clinical and preventive medicine using bibliometrics. This has included offering potent guidance for the development of related research on pharmaceutical bionanotechnology [6], nanotribology[9], cardiovascular and cerebrovascular diseases[10], tumor[11], health care[12], diabetes[13], and AIDS[14].

This study, for the first time, employed the method of bibliometrics to analyze intracranial aneurysm publications found on the Web of Science (WOS, Thomson Reuters Company) database from 1991 to 2012. Findings were analyzed to better understand trends in global research, contributions from Chinese authors, and characterize China's research status and regional (mainland China [ML], Taiwan [TW], and Hong Kong [HK]) differences.

## Materials and Methods

### Sources of the Data

Data were obtained from the Information Database Platform of Shanghai Institute for Biological Sciences, Chinese Academy of Sciences on 2013-08-15. The ISI WOS Citation and Essential Science Indications (ESI) databases were searched, including the Science Citation Index Expanded (SCIE), Conference Proceeding Citation Index-Science (CPCI-S), Current Chemical Reactions (CCR)-Expanded, and Index Chemicus (IC). Journal impact factor (IF) complied with the standard of ISI Web of Knowledge *Journal Citation Reports* 2012 database (2013-06-20). Foundation data were derived from the web of National Natural Science Foundation of China (NSFC).

### Search Strategy

#### Global Intracranial Aneurysm Research

Search terms were: Theme  =  ((intracranial aneurysm*) or (cerebral aneurysm*) or (brain aneurysm*)) AND publishing year  =  (1991–2012). Refining: literature type  =  (Article or Letter or Review).

#### Intracranial Aneurysms Research of China

Search terms were: Theme  =  ((Intracranial aneurysm*) or (cerebral aneurysm*) or (brain aneurysm*)) AND publishing year  =  (1991–2012); Refining: literature type  =  (Article or Letter or Review). For a comprehensive selection of research from China, the country/region was set as “Peoples R China or Taiwan”. Researches from different regions of China were also selected, and the refining conditions were set as: country/region  =  (Taiwan); address  =  (Hong Kong); and address  =  Peoples R China NOT Hong Kong.

### Data Collection

The txt data download from WOS were imported into Microsoft Excel 2007, and the data entry and collecting was verified by two authors. The final data were further cleaned and analyzed in Excel manually. Bibliometric indicators, including publication number, citation frequency, average number of citations per paper, h-index[15], [16], and support from science foundations were extracted from the data to quantitatively and qualitatively analyze the publications.

### Statistical Methods

Analyze Tool of WOS was used to analyze the characteristics of the publications, including time, country and region, authors, research institution, research orientation, and proportion of papers with foundation. Matlab software was used to fit the time trend of the publications. The logistic regression model: *f*(*x*) = *c*/ [1+*a*×*exp*(−*b*×*x*)] was used to model the cumulative volume of documentation due to its good fit and ability to predict future trends in the literature [17], [18]. The inflection point of the logistic curve was the time point when the publication growth rate moved from positive to negative. The formula used to generate the point was *T* = ln *a*/*b* [17], [18]. Thomson Data Analyzer (TDA) software was used to analyze the collaborations between countries and regions. EndNote X6 software was used to collect and analyze the highly cited articles. In order to investigate the Chinese research status, we compared the publications of USA, Japan, Germany, and China. We also compared the research from ML, TW, and HK.

## Results

### Global Cerebral Aneurysm Publication Trends

#### Growing Trends and Countries/Regions Contributing to Global Publications

**Figure 1:**
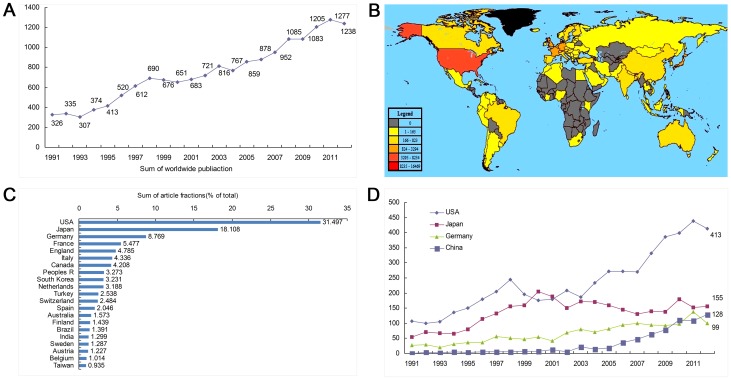
Countries/Regions Contributing to Intracranial Aneurysm Research. A: The time curve of worldwide intracranial aneurysm publications; B: World map showing the distribution of intracranial aneurysm publications; C: The sum of intracranial aneurysm research-related article fractions (% of research from each region) from the top 21 countries/regions; D: The time curve of intracranial aneurysm articles from the USA, Japan, Germany, and China.

There were 16468 articles meeting the search criteria from 1991 to 2012. Their cumulative cited frequency was 273500 times, cited frequency per paper 16.608 times and annual citations per paper 12 431.818 times. The global number of publications on intracranial aneurysms retrieved from SCIE increased from 326 in 1991 to 1238 in 2012. A marked increase was seen after the ISAT study published in 2002 (Figure 1A). A total of 155 countries and regions contributed to the world literature (Figure 1B). The United States published the largest number of studies of intracranial aneurysms (5187 published papers, 31.497%), followed by Japan and Germany. The publication numbers in the above three countries accounted for 58.374% of all publications (Figure 1C). Mainland of China (including Hong Kong) ranked eighth, accounting for 3.273%, and Taiwan ranked 21^st^, accounting for 0.935% (Figure 1C).

**Figure 2:**
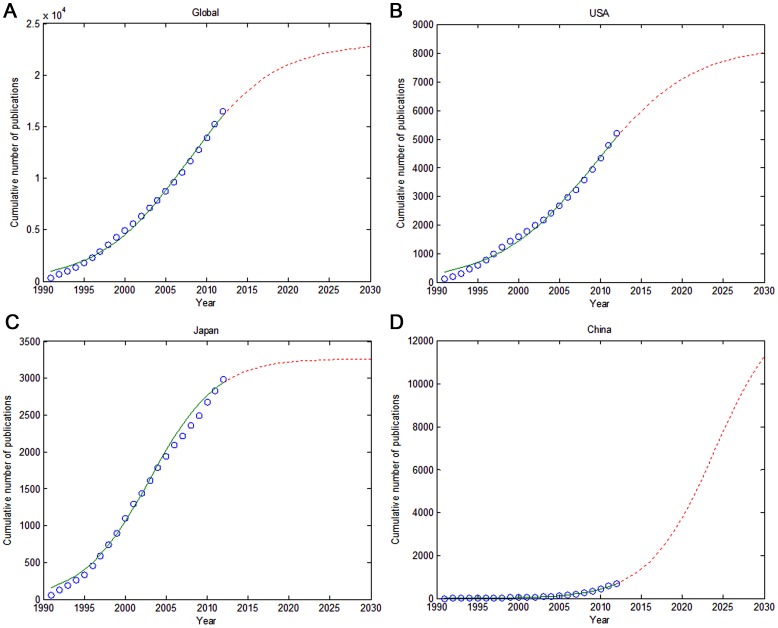
Model Fitting Curves of Growth Trends of Intracranial Aneurysm Publications. A: Global; B: USA; C: Japan; D: China.

The curve fit to model the global research paper output growth (Figure 2A) demonstrated an inflection point in 2008, the latter part of the logistic curve. Future literature growth was slower, but maintained a constant level. The inflection point came in 2009 in the United States (Figure 2B), 2003 in Japan (Figure 2C), and 2008 in Germany (data not shown).

#### Citation and H-index Analysis

**Figure 3:**
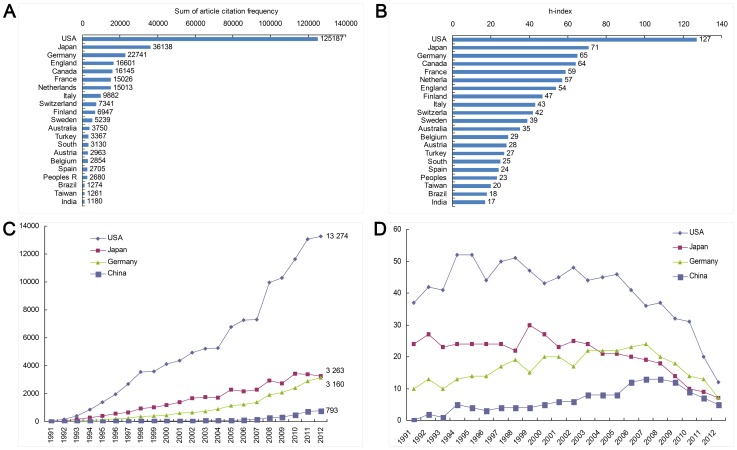
Citation Frequency (A, C) and H-index (B, D) of Different Countries/Regions over Time.

The number of citations of papers published in the United States was 125187, accounting for 58.64% of the total citations. There were 24.13 citations per paper (Figure 3A). The h-index of papers published in the United States (Figure 3B) was higher than that of other countries or regions, indicating that the United States led the world not only in quantity but also in quality of publications. Japan's contribution to research in this field was second highest, with a relatively high citation frequency (Figure 3A) and h-index (Figure 3B). The Netherlands and Finland did not have a large number of publications, but their citation frequency and h-index were relatively high, suggesting higher quality research.

#### Distribution of High Contribution Institutions/Authors and Research Orientations

**Figure 4:**
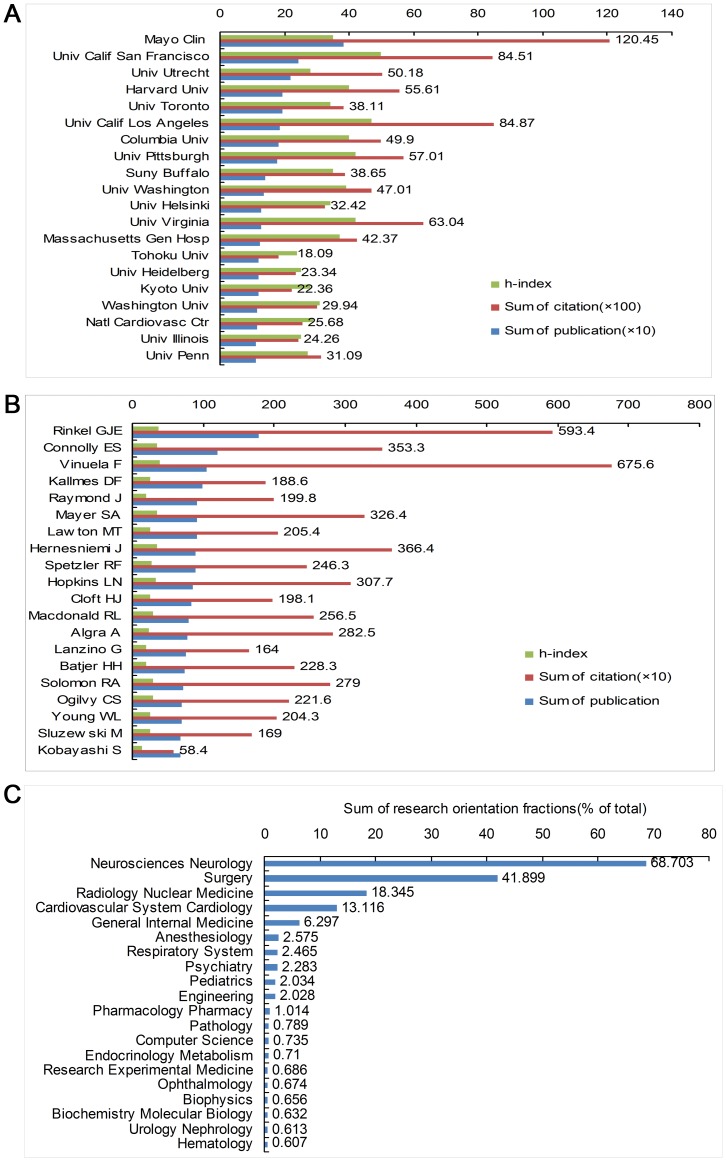
High Impact Institutions(A), Authors(B), and Research Orientation (C) of Intracranial Aneurysm Research.

7013 institutions from different nations or regions participated in aneurysm research. The top 20 contributing institutions are listed in Figure 4A. These 20 institutions accounted for 19.133% (3151/16468) of the articles published and 33.28% (91 012/273 500) of citations. The top 20 institutions were mainly derived from universities throughout the world. American colleges and universities dominated these, consisting of 65% (13/20) of the institutions. The remaining seven institutions were from Japan (3), Canada (1), the Netherlands (1), Germany (1), and Finland (1).

The author with the most publications was Professor Gabriel JE Rinkel from University Utrecht, publishing 179 papers related to aneurysms and being cited 5934 times. The highest cumulative citation frequency (6756) was from Professor Viñuela Fernando of the Division of Interventional Neuroradiology, UCLA Medical Center, Los Angeles, California, USA. Among the top 20 highest contributing authors (Figure 4B), there were 13 from United States, 3 from the Netherlands, 2 from Canada, and 1 from Finland, and 1 from Japan.

There were 50 fields of intracranial aneurysm research, with the Neurosciences Neurology, Surgery, and Radiology Nuclear Medicine Medical Imaging being the most common (Figure 4C).

#### Distribution of Published Journals on Intracranial Aneurysm

Publications occurred in 1362 different journals from 1991–2012. 23 journals were published ≥5 papers yearly, accounting for 51.985% of the total number. 58 journals were published ≥2 papers yearly, accounting for 66.589% of the total number. The remaining 1304 journals were only published 1 paper a year. The journals with the highest publication number and highest total citation rate were *Neurosurgery* (IF 2.532), *Journal of Neurosurgery* (IF 3.148), *American Journal of Neuroradiology* (IF 3.167), *Stroke* (IF 6.158), and *Act Neurochirurgica* (IF 1.546).

### Analysis of Chinese Research of Intracranial Aneurysm

#### Growing Trend of Chinese Publications and Comparison to Global Publications

China published 692 intracranial aneurysm papers from 1991 to 2012. The number of published articles increased from 0 in 1991 to 128 in 2012. The increase in publication rate began in 2003. Chinese publications ranked fourth globally in 2009 and third in 2012. The number exceeded that of Germany and was followed that of the United States and Japan. The tendency in growth rate was such that the number would soon equal those from Japan (Figure 1D). Chinese research papers on intracranial aneurysm were at the early stage of the logistic growth curve. The publication rate appeared as an exponential growth (Figure 2D), and at the current time, appeared to be a long distance from the inflection point (2024). 60 papers were published from 1991–2001 (8.67%) and of the remaining 632 were published from 2002–2012 (91.33%).

#### Global Growth Trend of Citations and the H-index

The total citation rate of papers on intracranial aneurysm in China during 1991–2012 was 3954 times, the average citation rate was 5.71, h-index was 27, and the average citation rates were 197.7 times annually. Papers were cited 204 times from 1991–2001 and 2002 times (94.84%) from 2002–2012. The cited frequency in China started to rise in 2007, but was lower than that of the United States, Japan, and Germany (Figure 3C). These findings suggest that China lacked intracranial aneurysm research of high quality.

Between 1991 and 2012, the highest h-index was from the United States, followed by Japan and Germany. China ranked at the bottom. This indicated that, compared to advanced countries, the quality of Chinese intracranial aneurysm publications was low. The overall trend was one of improvement (Figure 3D).

#### Publication Journals

From 1991 to 2012, Chinese published papers in 160 different journals, without any concentration in any particular journals. The papers were mainly published in *Journal of Clinical Neuroscience*, *Interventional Neuroradiology*, *Chinese Medical Journal*, *Surgical Neurology*, and *Acta Neurochirurgica*, which shared a generally lower IF. China published dramatically more papers in the international aneurysm “core journals” from 2002–2012. In 1991–2001, *Chinese Medical Journal* contained 16.67% of all publications, while only 3.96% were published in this journal during 2002–2012. Since 2002, papers began to be published in *Stroke* (4) and *Radiology* (6), suggesting that the research level in China was improving.

#### Main Contributing Institutions and Funding Trends

**Figure 5:**
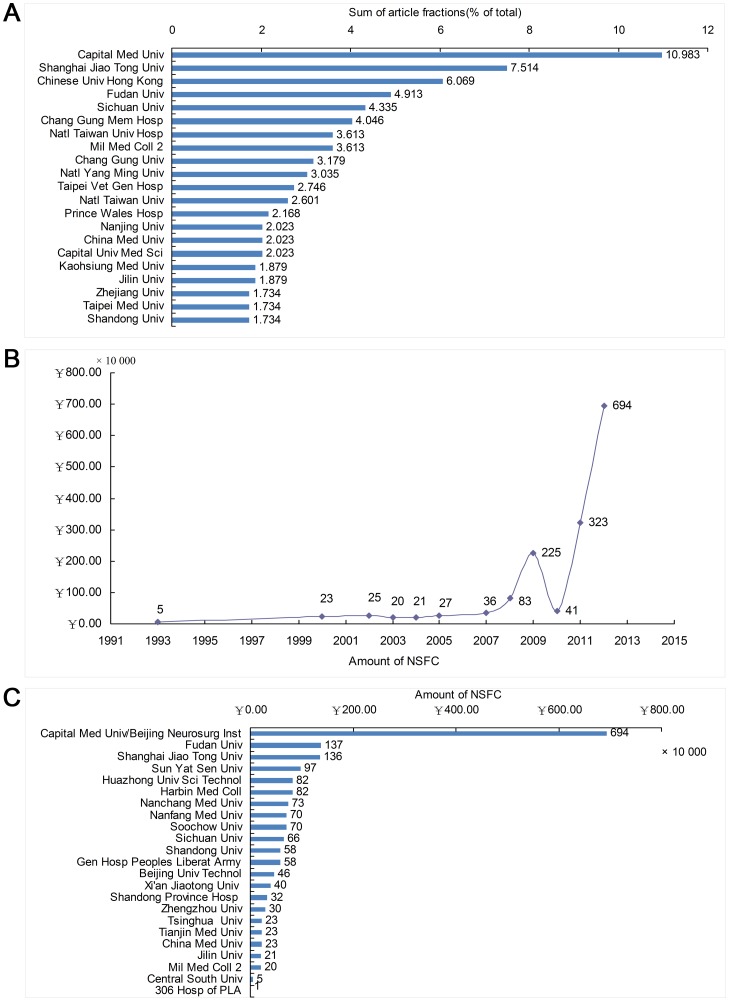
Contributing Institutions and Growth of NSFC Funding in Intracranial Aneurysm Research in China. A: Major institutions with intracranial aneurysm research in China; B: The time curve for NSFC total funding; C: NSFC funding of intracranial aneurysm research at different institutions.

370 Chinese institutions published intracranial aneurysm research from 1991 to 2012. The main contributors were Capital Medical University (10.98%, the largest contribution organization), Shanghai Jiao Tong University, Chinese University of Hong Kong, Fudan University, Sichuan University, Chang Gung Memorial Hospital, and the Second Military Medical University (Figure 5A). 339 Chinese intracranial aneurysm publications were supported by 138 funding institutions, a funding ratio of 48.99%. Support came mainly from the state, provinces and municipalities, schools, and equipment companies. The largest increase in funding occurred after 2007. The largest support came from the NSFC of China (21%), National “863”and (or) “973”Plans (11%), and Shanghai (19%) and Beijing municipalities (6%).

The NSFC was the main Chinese funding organization. The fund supplied 18.87 million RMB for intracranial aneurysm research from 1993 to 2013, with an increasing amount each year. Only about 50000 RMB was given in 1993. 230000 RMB was given in 2000, with further increases starting after 2002. Funding reached 6.94 million RMB in 2012 (Figure 5B). 23 institutions received NSFC funding. Capital Medical University received the majority of funding (6.94 million RMB, 36.78% of the total), followed by Fudan University (1.37 million RMB, 7.26%), and Shanghai Jiaotong University (1.36 million RMB, 7.21%) (Figure 5C).

#### Global Collaborations in Intracranial Aneurysm Research

**Figure 6:**
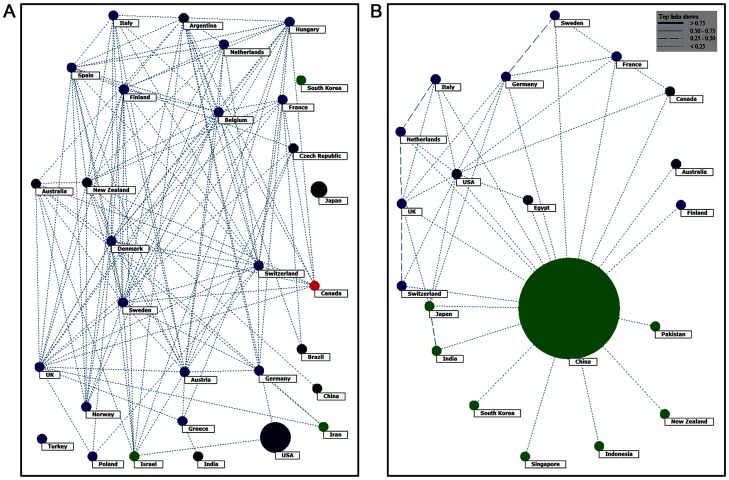
Intracranial Aneurysm Research Collaborations Globally (A) and in China (B).

The United States and Japan ran relatively independent studies, having less cooperation with other countries. European countries, Canada, and Australia tended to perform international multi-institutional studies (Figure 6A). China, India, and South Korea demonstrated less international collaboration. China demonstrated a low level of international collaboration. The main collaborating countries were the United States, Britain, Germany, France, Canada, and Italy. There were few Chinese collaborating with Japan or India (Figure 6B).

### Comparison of Intracranial Aneurysm Research in ML, TW, and HK

#### Comparison of Publication Number

**Figure 7:**
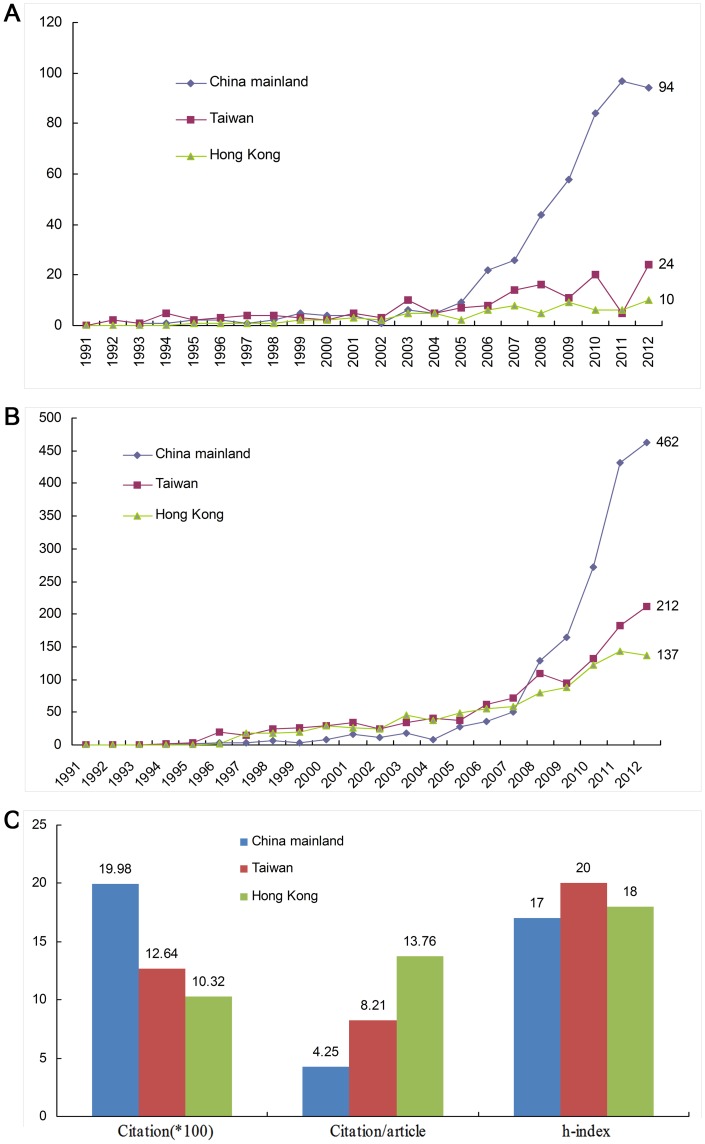
Comparison of Intracranial Aneurysm Research in Mainland China, Taiwan, and Hong Kong during 1991–2012. A: Number of papers; B: Citation frequency; C: Cumulative citation frequencies, citation frequency per article, and h-indices.

There were 470 ML publications, 154 from TW, and 75 from HK during 1991 to 2012. Intracranial aneurysm research in China gradually increased during this time period. The main increases were seen in ML in 2005 (Figure 7A).

#### Comparison of Citation Frequency

Number of publications and citation frequency gradually rose from 1991 to 2012 in all three regions. The citation frequency from ML increased rapidly after 2008, exceeding that of TW and HK (Figure 7B). The average citation frequency and overall publication number from ML was less than that from TW and HK. The citation frequency of intracranial aneurysm publications from ML was the highest in 1998, but was associated with the lowest average citation rate per article (4.25). HK had the lowest cumulative citation frequency (1032), but the highest average citation rates per year (13.76). TW had 1264 cumulative citations, 8.21 average citations per year, and the highest h-index, 20 (Figure 7C).

#### Comparison of Publications in the Core Journals

We evaluated the top 10 international publications based on number of publications and compared the publications in the three areas (Table 1). 107 (22.77%, 107/470) ML, 46 (29.87%, 46/154) TW, and 34 (45.33%, 34/75) HK papers were published in core journals. HK had the greatest proportion of high quality papers.

**Table 1:** Publications from different regions of China in top 10 core journals.

Journals	Mainland China	Taiwan	Hong Kong
*Neurosurgery*	6	6	4
*Journal of Neurosurgery*	12	9	4
*American Journal of Neuroradiology*	11	3	4
*Stroke*	3	1	3
*Acta Neurochirurgica*	11	9	7
*Surgical Neurology*	21	9	3
*Interventional Neuroradiology*	33	7	4
*Neuroradiology*	9	1	1
*Neurologia Medico Chirurgica*	1	1	0
*Neurological Surgery*	0	0	4

#### Comparison of International Collaborations

There were 18 countries or regions collaborating with ML in the research of intracranial aneurysm, accounting for 19.362% of the total publication number (91/470). The main cooperating organizations were located in the United States, Japan, Germany, and France. Ten countries or regions (24.672%) worked in cooperation with TW. The main cooperating organizations were the United States, France, and Japan. 11 countries and regions (21.331%) performed research with HK. The main collaborators were New Zealand and France.

## Discussion

### Trends in Global Intracranial Aneurysm Research

The global trend in intracranial aneurysm research has increased since 1991, especially after the ISAT findings published in 2002. The cumulative amount of literature followed a logistic growth model, with the inflection point in 2008. The growth is now in the latter part of the logistic growth curve, characterized by slow steady growth for about 10 years. The major contributors to research, in descending order, were the United States, Japan, Europe (Germany, France, Britain, and Netherlands), Australia and Canada. The most highly cited papers, with the most influential authors and the greatest funding came from the United States. Japan was second in these parameters, although they did exceed the United States once in 2000. There has not been much recent growth since the inflection point in 2003. While Europe Germany, France, Britain, the Netherlands, Australia, and Canada published fewer papers than the United States and Japan, they did publish many influential Randomized Controlled Trials (RCT) studies, including the ISAT study (1218 citations up to 2013-08-15). European countries used an integrated research platform with collaboration between countries. Research in Asian countries (China, South Korea, and India) increased markedly, although the overall level of research was relatively low. The journal distribution of the publications was in line with Bradford's law [19]. Specialized journals do not have high impact factors. The highest IF was held by *Stroke*, 6.158. Several highly cited papers were published in more generalized journals, such as the *New England Journal of Medicine*, *the Lancet*, *Science*, and *Nature*.

### Growing Trend in China's Contribution to Intracranial Aneurysm Research

Intracranial aneurysm research in China trailed the world trend, with few publications before 2001. The number of publications increased rapidly after 2002, exceeding Germany in 2012. The anticipated inflection point appears to be in 2024.

China's rapid development in the research of intracranial aneurysm may be largely attributed to: (1) Overall improvement in economic level, science and technology strength in the last 30 years. The latest WOS data (2013-06-20) showed that the quantity and quality of Chinese SCI papers has continued to increase. The quantity of the international papers published was second only to the United States. The citation frequency of Chinese papers ranked sixth. Clinical and basic medicines were the most productive SCI disciplines in China, list 2^nd^ and 6^th^, respectively. (2) With the improved economy, many hospitals in China have become equipped with modern diagnostic and treatment devices for intracranial aneurysm (including 3-D DSA, MRA and CTA, PET-CT, stent, coil, and flow diversion). (3) China's overall basic research level is increasing [5], with access to the latest experimental techniques, reagents, and equipment. The use of transitional medicine and multidisciplinary cooperation are increasing, improving the basic research of intracranial aneurysm. (4) Increased governmental funding has enhanced health care insurance[20], enabling more patients to afford medical care and increasing access to treatment. This funding has included research on intracranial aneurysm, allowing more investigators and institutions to conduct research. (5) The improvement of living standards has led to longer life and an increase in the number of cerebrovascular patients with aneurysm in China[21], [22]. The improvement in medical technology has increased the ability to diagnose aneurysm, making it possible to obtain timely treatment. China's large population is associated with a number of the aneurysm patients to treat and study. (6) The current scientific evaluation system is based on Science Citation Index papers in China[23]. This motivates researchers to participate in high quality research regarding intracranial aneurysm.

The quality of intracranial aneurysm research in China is generally low. China lacks high quality RCTs and international cooperation is at a low level.

### Comparison of Intracranial Aneurysm Research from ML, TW, and HK

Owning to historical reasons, the research strength level in TW and HK is higher than that of ML. This trend is seen in many other disciplines[24]–[26]. In order to define the regional differences in China, we made a comparative analysis of research from ML, TW, and HK. The number of intracranial aneurysm publications and citation frequency from ML were obviously higher than that from TW and HK. The number of citations per paper and per cent of publications occurring in high impact journals were lower in ML. ML is a major contributor to the study of intracranial aneurysm, followed by TW and HK. The reasons may be that: (1) The period of rapid development of intracranial aneurysm study has been relatively short, mostly occurring after 2002. (2) ML put large amounts of funding into intracranial aneurysm research, much more than TW and HK. (3) The patient population is larger in ML, which provides a larger sample for related research, and (4) There is a larger number of researchers in ML, who are involved in intracranial aneurysm research. Overall, ML, TW, and HK have limited research collaborations; TW and ML had only one collaborative publication.

### Strengths and Limitations

International medical journals evaluated in this study were reviewed from the WOS database of SCIE journals. The data analysis was relatively comprehensive and objective. However, the SCIE database was mainly based on the English literature and many non-English publications were not included. A large portion of the excluded literature was Russian and Chinese. Further, this was a global analysis, and was not adjusted for population size. Future work including other languages and adjusting for population characteristics would refine these results.
